# Application of Quantum Computing to Biochemical Systems: A Look to the Future

**DOI:** 10.3389/fchem.2020.587143

**Published:** 2020-11-24

**Authors:** Hai-Ping Cheng, Erik Deumens, James K. Freericks, Chenglong Li, Beverly A. Sanders

**Affiliations:** ^1^Quantum Theory Project, Department of Physics, University of Florida, Gainesville, FL, United States; ^2^Department of Physics, Georgetown University, Washington, DC, United States; ^3^Department of Medicinal Chemistry, University of Florida, Gainesville, FL, United States; ^4^Department of Computer and Information Science and Engineering, University of Florida, Gainesville, FL, United States

**Keywords:** computational molecular biology, biochemistry, quantum computing, hybrid quantum-classical algorithms, quantum embedding theory

## Abstract

Chemistry is considered as one of the more promising applications to science of near-term quantum computing. Recent work in transitioning classical algorithms to a quantum computer has led to great strides in improving quantum algorithms and illustrating their quantum advantage. Because of the limitations of near-term quantum computers, the most effective strategies split the work over classical and quantum computers. There is a proven set of methods in computational chemistry and materials physics that has used this same idea of splitting a complex physical system into parts that are treated at different levels of theory to obtain solutions for the complete physical system for which a brute force solution with a single method is not feasible. These methods are variously known as embedding, multi-scale, and fragment techniques and methods. We review these methods and then propose the embedding approach as a method for describing complex biochemical systems, with the parts not only treated with different levels of theory, but computed with hybrid classical and quantum algorithms. Such strategies are critical if one wants to expand the focus to biochemical molecules that contain active regions that cannot be properly explained with traditional algorithms on classical computers. While we do not solve this problem here, we provide an overview of where the field is going to enable such problems to be tackled in the future.

## 1. Introduction

Biochemical systems are essential for carrying out biological functions, and their actions span extreme time and length scales. These systems consist of proteins, DNAs, RNAs, carbohydrates, or lipids (either individually or in combination) with small molecule ligands and/or with ions in aqueous or membrane environments. The functional processes can be either covalent or non-covalent, such as molecular recognition; or a combination of both, such as an enzymatic cycle. Important biological functions are, for example, stem cell maintenance, DNA repair, gene transcription and translation, signal transduction, development, learning and memory, metabolism, etc. In order to understand these elementary processes, together with experimental approaches, various computational methods have been developed at the electronic, the atomic, and more coarse-grained levels over the decades. However, full quantum calculations are intractable due to the large molecule sizes and the high demands for accuracy required for chemical applications.

The solution may lie in quantum computing: as Feynman once said, “. Nature isn't classical.if you want to make a simulation of Nature, you'd better make it quantum mechanical.” (Feynman, 1982). As a matter of fact, from the remarkable speed of enzyme-catalyzed reactions to the workings of the human brain, numerous biological puzzles are now being explored for evidence of quantum effects. Well-known examples include photosynthesis, nitrogen fixation, magnetoreception, olfaction, neuronal signal processing, protein/drug interaction, and so on. There have even been early attempts to develop quantum computing algorithms specifically for nitrogen fixation (Reiher et al., 2017).

Quantum computing is being explored to help solve a variety of problems in biochemistry and biology (Cao et al., 2019; Emani et al., 2019). In this paper, we review several approaches to allow quantum computing to be exploited to simulate biochemical systems with complicated electron correlation. We formulate a general approach of embedding to describe part of the system on classical computers and the most demanding part on a quantum computer resulting in a complete solution of the complex system with useful accuracy. This will allow quantum computers to be used for such demanding problems without the requirement that a quantum computer be available to hold and process the entire system of interest. In section 2, as motivating examples, we present three biochemical systems that are intractable with classical algorithms on classical computers due to the need to deal with complicated electron correlation. Effectively addressing them with quantum computing will lead to important scientific advances. Then we review the embedding methodologies that have been used to handle very complex systems using classical computers in section 3, which consists of dividing the system into two parts, with one part, the most computationally demanding, computed with quantum theory and the other part, considered the environment, treated with classical theory. The challenge of embedding methods is the exchange of information between the two parts. Section 4 provides a brief review of two of the most important existing quantum algorithms for chemistry, Variational Quantum Eigensolver and Quantum Phase Estimation. Section 5 presents how the idea of embedding strategies can be usefully applied to handle complex physical systems at a high level of accuracy by combining the power of quantum computers for the strongly correlated part of the system with the use of classical computers for the other parts.

In the context of computational chemistry, the distinction of quantum vs. classical has two meanings that are both relevant: The first, traditional, meaning designates the level of theory that is used to describe the chemical and biochemical systems. Because of the complexity and size of biochemical systems, treating the whole system using quantum theory is not feasible and often the systems are described using theories based on classical physics. The second, more recently introduced, meaning refers to whether the theoretical model and computational algorithms are run on quantum computers or classical computers. The promise of quantum computers is that they will eventually be sufficiently powerful to allow scientists to model complex biochemical systems accurately and efficiently with a fully quantum theoretical description. In this paper, we will make it clear which meaning is used when it is used.

## 2. Motivating Examples

We present three important and representative biochemical systems whose properties make them attractive targets for quantum computing. The first two are open shell transition-metal and conjugated pi-electron strongly correlated systems; the last one displays extreme non-covalent intermolecular binding involving a large number of atoms. These three examples symbolize difficult cases for classical quantum chemical treatments and superior ones for quantum computing.

### 2.1. A Transition-Metal-Ion-Containing Enzyme: Histone Demethylase

The transcription of genetic information encoded in DNA is in part regulated by chemical modifications to histone proteins. Histone demethylases are enzymes that remove methyl (-CH3) groups from histones. The demethylase proteins alter transcriptional regulation of the genome by controlling the methylation levels that occur on DNA and/or histones and, in turn, regulate the chromatin states at specific gene loci within organisms. The big demethylase family has KDM1-6 classes (Pedersen and Helin, 2010). Defined by their mechanisms, two main classes of histone demethylases exist: a flavin adenine dinucleotide (FAD)-dependent amine oxidase, and an Fe(II) and α-ketoglutarate-dependent hydroxylase. Both operate by hydroxylation of a methyl group followed by dissociation of formaldehyde. By studying various demethylation details, improvements are possible in the understanding of how “histone code” is employed for gene on/off switching.

**Figure 1:**
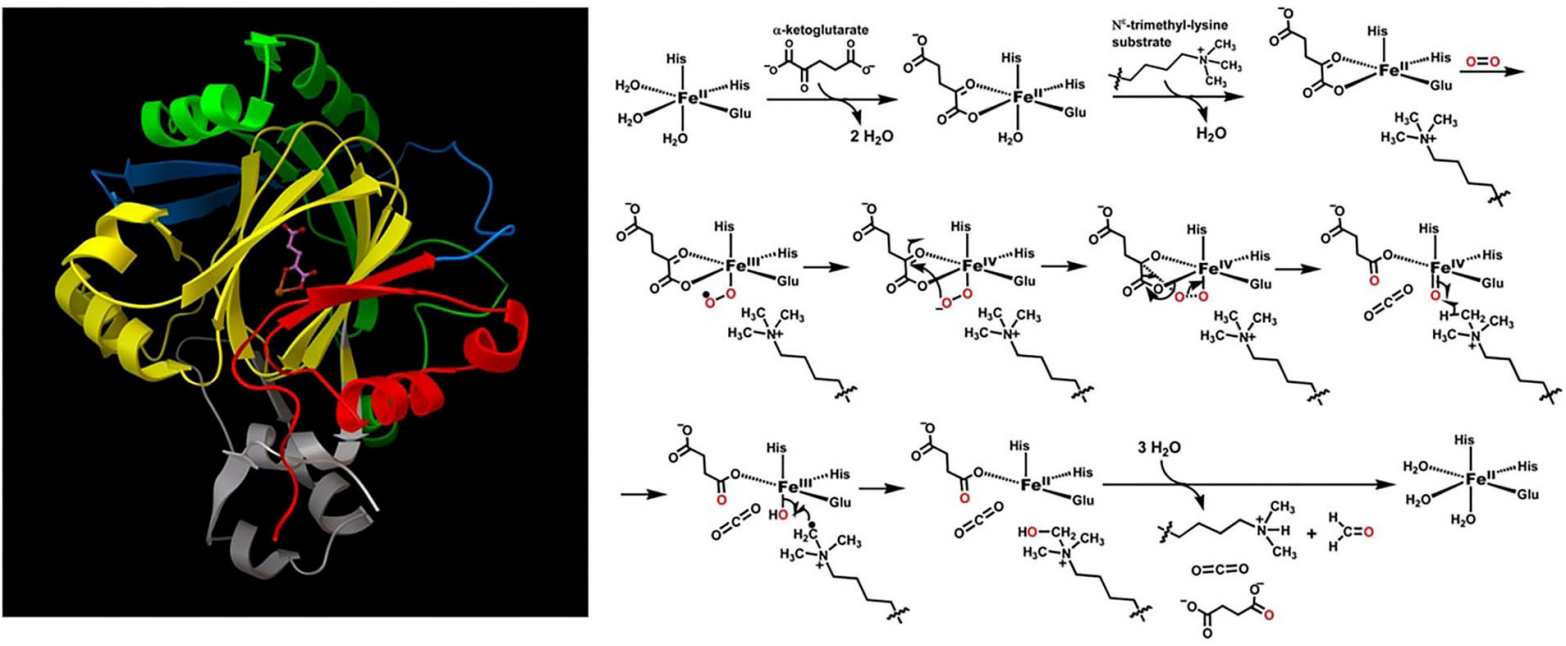
Structure of JmJD2A. Some domains from above are highlighted: JmJ (N-terminus, red; C-terminus, yellow), Zinc finger domain (light purple), Beta-hairpin (light blue), and mixed domain linker (green). The ball-and-sticks are Fe(II) and alpha-ketoglutarate cofactors. The enzymatic reactions involve both iron redox and oxygen radical, and are thus infeasible with classical computers.

Figure 1 is an illustration of the JmJD2A topology, active site, and proposed catalytic mechanism which involve both transition metal ions and reaction radicals (Chen et al., 2006; Ng et al., 2007; Zheng and Huang, 2014). These are cases where the Born-Oppenheimer approximation breaks down. During the catalytic cycle, the iron metal ion has three charge states: +2, +3, and +4, and two spin states: 0 and 1/2. Oxygen has three spin states: 0, 1/2 and 1. There are at least nine catalytic steps. Considering only direct contact catalytic amino acid residues, oxygen, trimethylated quaternary amine from lysine substrate, and of course catalytic Fe ion, 151 electrons and 121 spatial orbitals must be involved to achieve accurate electronic structure and related energy calculations.

### 2.2. Non-Metal-Ion-Containing Enzyme: Telomerase

**Figure 2:**
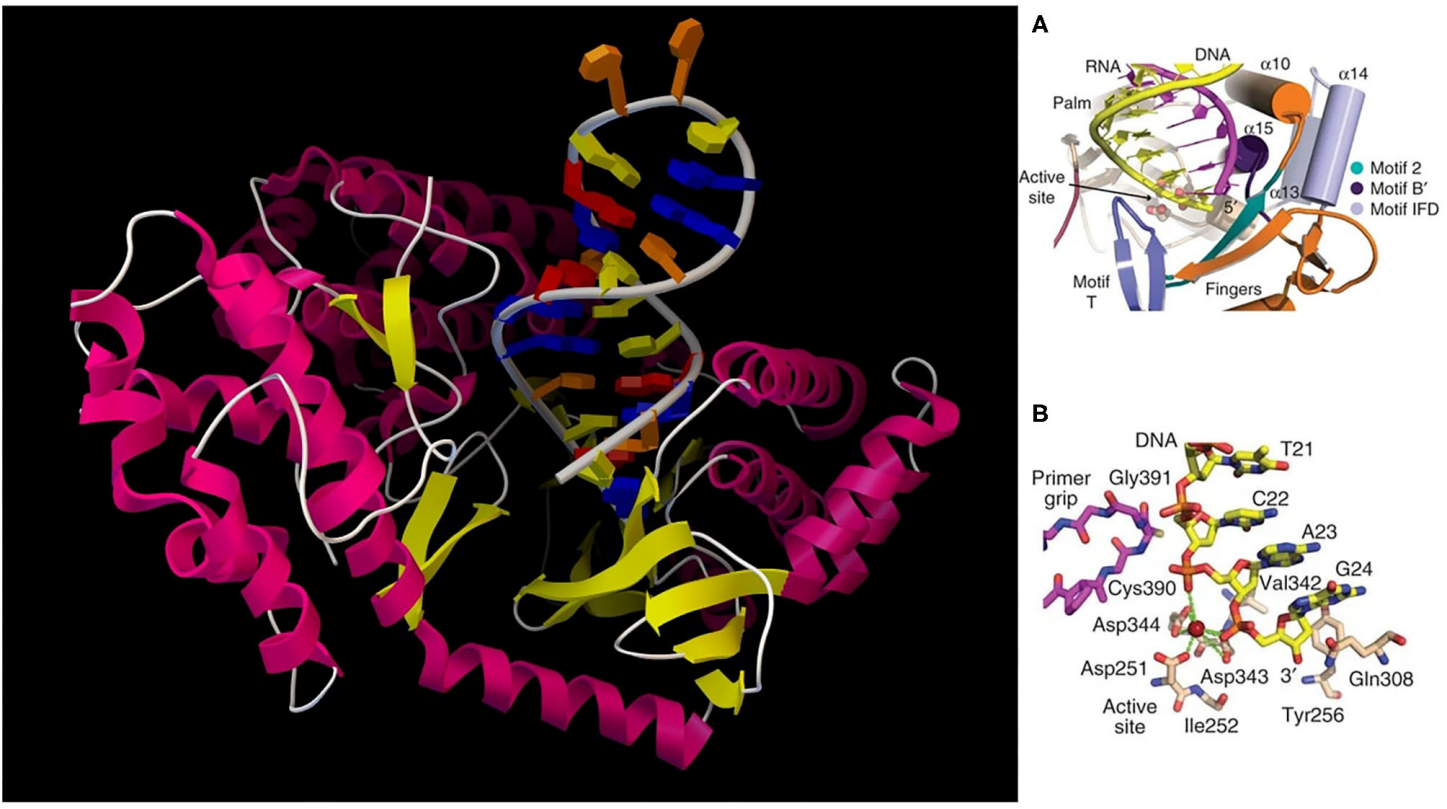
(Left) TERT with hybrid RNA/DNA bound; (Right) **(A)** cartoon representation of the active site; **(B)** detailed active site residues and DNA substrate.

The study of telomerase is of tremendous significance for understanding stem cell maintenance, aging, and cancer. At each end of a chromosome, there is a region of repetitive nucleotide sequences called a telomere which protects the chromosome from deterioration or fusion with neighboring chromosomes. During chromosome replication, the enzymes that duplicate DNA cannot continue their duplication all the way to the end of a chromosome, so after each cell division, the telomere gets shorter. Telomeres are replenished by an enzyme, as shown in Figure 2, telomerase reverse transcriptase (TERT), (Cohen et al., 2007) which is the catalytic subunit of telomerase. Telomerase is active in normal stem cells and most cancer cells, but is normally absent from, or at very low levels in, most somatic (body) cells.

For vertebrates, the sequence of nucleotides in telomeres is AGGGTT (Harvey, 2014). The complementary DNA strand is TCCCAA, which also has a single-stranded TTAGGG overhang (Witzany, 2008). This sequence of TTAGGG is repeated approximately 2,500 times in humans (Sadava et al., 2011). The active telomerase is a homodimer, each monomer having telomerase reverse transcriptase (TERT), telomerase RNA, and dyskerin (Mitchell et al., 2010). Currently, there are several TERT crystal structures available; computational simulation of TERT telomere elongation is important. Snapshots of the molecular processes need be constructed and quantum computing could be used to simulate the catalytic active centers in order to better understand how these systems work, especially base fidelity preservation during the extension process. Due to its nucleobase pairing and reaction processivity, this is a case where quantum computing could make a large impact on molecular recognition.

### 2.3. Molecular Recognition: Biotin-(Strept)Avidin Binding

**Figure 3:**
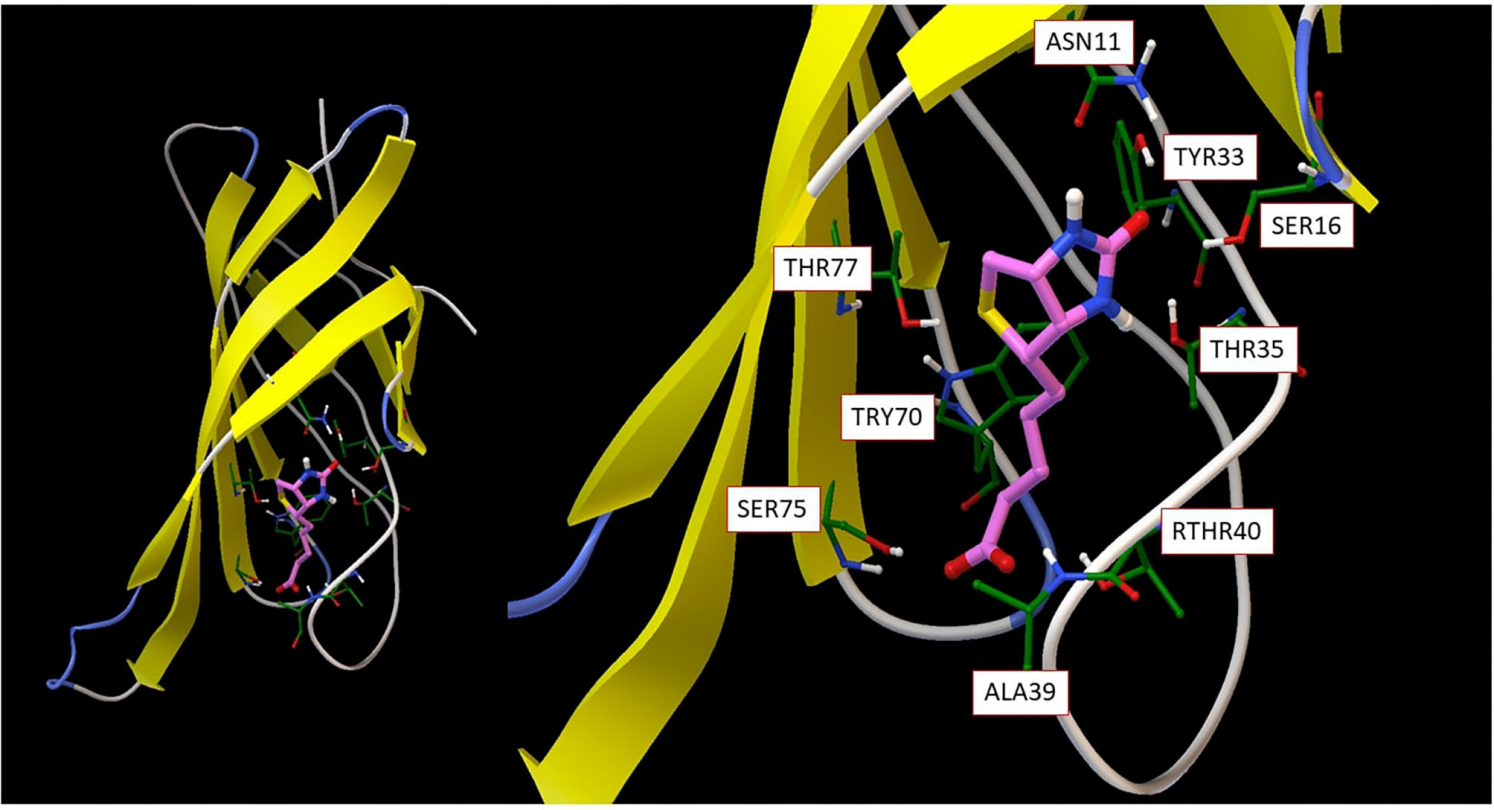
**(Left)** overall avidin protein structure with biotin binding; **(Right)** detailed biotin interaction amino acid residues from avidin.

Molecular recognition, the specific interaction between multiple molecules which exhibit molecular complementarity through non-covalent binding, plays a critical role in biological interactions. Although the field is well-studied, important problems remain unsolved. For example, even though it is a classic molecular recognition issue and many studies have attempted to resolve it, the origin of strong non-covalent reversible binding of small molecule biotin to proteins avidin (Ka ~ 10E15 M-1) and streptavidin (Ka ~ 10E13 M-1) remains a mystery. As seen from Figure 3, the beta-barrel shaped avidin binds the biotin ligand with van der Waals, electrostatic, hydrogen bonding and pi-electron polarization forces; this results in a free energy of binding around −20 Kcal/mol, almost at a quasichemical bonding level.

Understanding this binding thermodynamics at the molecular level holds fundamental importance theoretically and offers key insights for molecular design. There is no doubt that in desolvation, conformational and vibrational entropy play an important role. However, the key issue here is to understand biotin-avidin intermolecular interaction, which rests on computing accurate non-bonded interaction energies. The biotin molecule (as the ligand) has 89 valence electrons and 79 frontier orbitals. By only considering direct contacting atoms from the binding amino acid residues, the active part of the molecule has a total of 379 electrons and 358 spatial orbitals. The spin state is *S* = 0 and charge state is −1. It should be obvious that such a subsystem is too large to be tackled with standard chemical methodologies for strongly correlated molecules, so quantum computing is the only option for a complete theoretical analysis. Sometimes, ligands form covalent bonding with proteins, such as anticancer drug ibrutinib binding to Bruton's tyrosine kinase (Bender et al., 2017). Quantum computing mimicking these processes not only helps fundamental understanding on molecular recognition, but also facilitates drug or materials design.

## 3. Classical Computer Embedding Strategies

In this section, we review the methodology of embedding as it has been used for several decades to describe complex chemical systems, including large bio-molecules in liquids and solid-state systems, by dividing them into parts that are treated with different levels of physical theory. The levels of theory range from continuum models, to classical dynamics of atoms and molecules, to full quantum-mechanical description of electronic structure and nuclear motion. These methods are then implemented in algorithms that run on classical computers. The challenge common to all these methods lies in the description of the interactions across the boundaries between the parts. There the interaction must be described with care because of the different theories being used to describe the parts on either side of the boundary.

The same methodology used to divide complex systems into parts is extended to describe some parts with algorithms that are executed on classical computers, while other parts are described by algorithms that are executed on quantum computers. The challenge of embedding methods on classical and quantum computers is the same in that the description of the interaction between the parts running on the classical and quantum computers must be handled with extreme care. In addition, because one cannot directly readout the final wavefunction from the quantum computer, hybrid algorithms must be properly designed to allow for the information from the quantum calculation to be transferred to the classical algorithm and *vice versa*.

In the method of multiscale simulation, the challenge is to describe the processes that are visible at the macroscopic level, but are fully determined by the details at some microscopic level. A paradigmatic example is the formation and propagation of cracks in materials (Gao and Klein, 1998; Rudd and Broughton, 2000; Rountree et al., 2002; Liu et al., 2004; Budarapu et al., 2014; Talebi et al., 2014). The macroscopic description is the goal, but continuum models that are effective and affordable at that scale cannot describe the basic-bond breaking process that lie at the foundation of the crack formation. Nor can molecular dynamics methods describe this process. Thus the continuum model, the molecular dynamics model, and the quantum model must all be coupled together with scale-bridging techniques used to connect them in a way that accurately preserves the physics (Hoekstra et al., 2014).

Similarly, a biomolecule can be divided into three regions: a classical region where interatomic interaction can be treated with classical force fields using standard methods (Amber http://ambermd.org/, CHARMM https://www.charmm.org/charmm/, LAMMPS https://lammps.sandia.gov/, etc); a quantum region where mean-field approximations are sufficient; and a strongly correlated region where high-level methods that treat quantum entanglement are needed, that is, techniques beyond density functional theory (DFT).

### 3.1. Hybrid Quantum-Classical Molecular Dynamics

**Figure 4:**
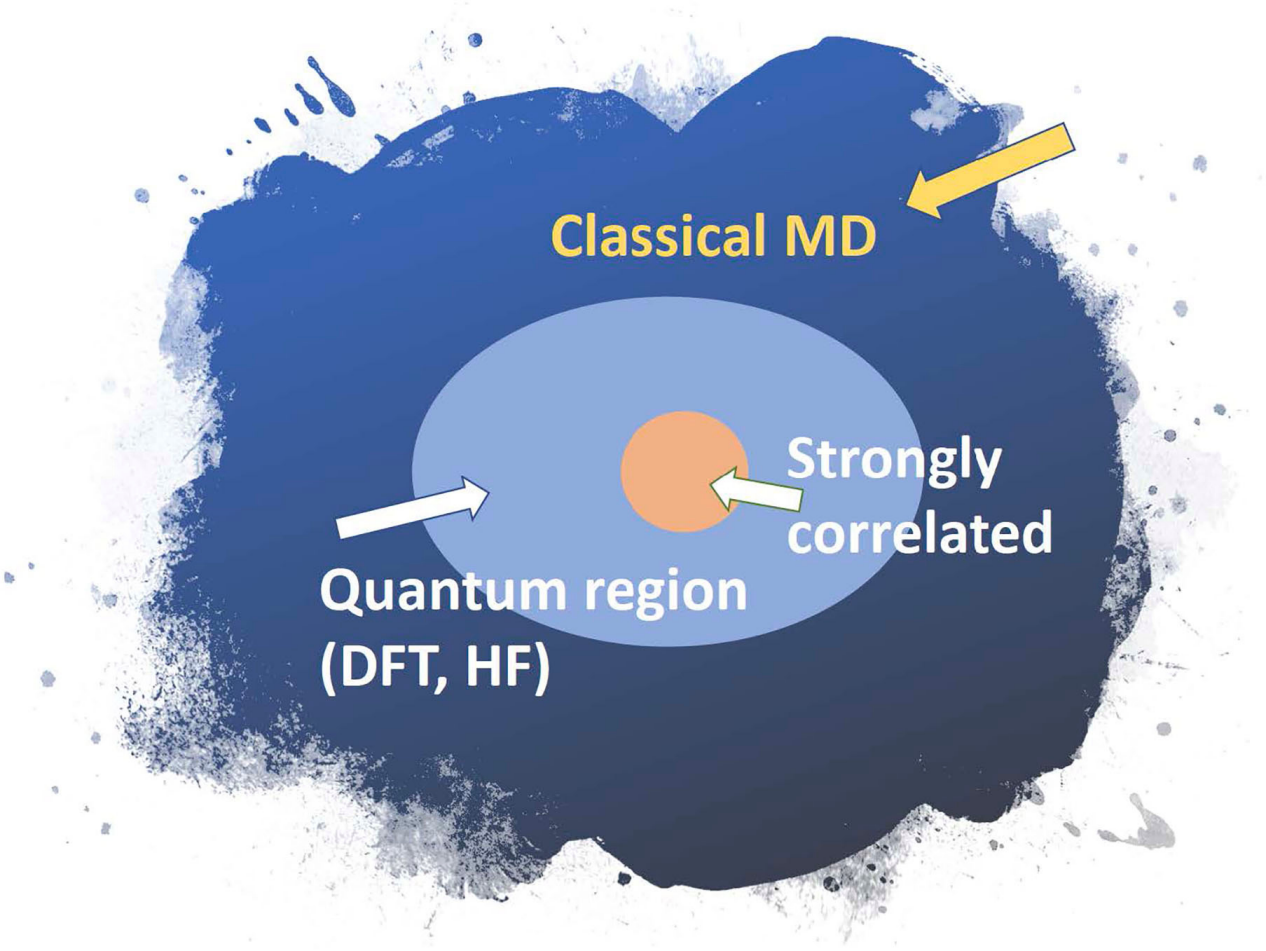
Schematic of a hybrid simulation framework for molecules that employs a hierarchical embedding strategy.

**Figure 5:**
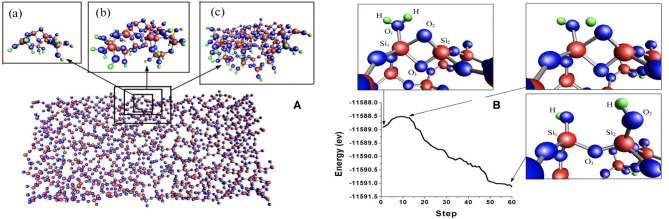
**(A)** Sketch of the hybrid simulation framework as applied to amorphous glasses from Du et al. (2004). Three sizes of the quantum region were chosen (panels a–c on the left) to ensure the convergence of the reaction energy. **(B)** The right picture relates energy path with water splitting process. The energy barrier is only 0.4 eV and is zero when the reaction involves only two water molecules.

We illustrate how hierarchical methods have been used in a few studies. We show a study of water-silica surface interaction, which shows that very complex amorphous systems can be handled by the method. In the amorphous water-silica interface interaction, the system is divided into two regions, the quantum and the classical. Here, the quantum region is described by effective mean-field methods, while the classical region is described by molecular dynamics using effective force laws. The two regions must be coupled together across their boundary. Various methods exist for the embedding of the quantum region (the light blue region in Figure 4) inside the classical region. In earlier work (Du et al., 2004), a quantum region described by DFT is embedded in a classical matrix as shown in Figure 5. This figure depicts a Si-O bond-breaking process on the silica surface. According to a free cluster model (Walsh et al., 2000), the calculated barrier energy of this process is *E*_*b*_ = 0.7–1.1 eV. However, when the cluster is embedded in a surface matrix, the calculated barrier energy *E*_*b*_ is equal to 0.4 eV. Including quantum effects results in a substantial decrease.

For a bio-molecule, the embedding is simpler than for amorphous materials as there are not as many bonds that connect the classical region and the quantum region. Techniques for this type of embedding are quite sophisticated (Gao and Xia, 1992; Bakowies and Thiel, 1996; Gao et al., 1998; Cui et al., 2001; Laio et al., 2002; Vreven et al., 2003; Friesner and Guallar, 2005).

The peptide hydrolysis reaction mechanism for HIV-1 protease has been studied by a hybrid Car-Parinello/classical molecular dynamics simulation (Piana et al., 2004)/Gradient-corrected BLYP density functional theory describes the reactive part of the active site and the AMBER force field describes the rest of the proteins, the solvent, and the counterions that are needed to balance the QM/MM description. The authors find that the orientation and the flexibility of the reactants, determined by the embedding protein structure, are important in determining the activation barrier for the reaction. This shows the need to include the larger structure as well as the ability of the QM/MM approach to address the problem.

As another example, a recent study (Ahsan and Senapati, 2019) on the effect of water, i.e., hydrogen bonds, in a catalytic role in epoxide ring opening in aspartate proteases using QM/MM show that the process follows a two-step mechanism with the formation of an oxyanion intermediate, which is stabilized with up to 30 kcal/mol supplied by the hydrogen bonds from the water molecules near the protein active site. This example, too, illustrates that the treatment of the whole system is crucial for a correct understanding of the biochemistry enabled by the hybrid approach.

### 3.2. From DFT to Strongly Correlated Systems: Quantum Embedding Theory for Molecules and for the Hubbard Model

Transition-metal molecular complexes with *d* and *f* electrons often demonstrate strong correlation effects. Active centers of many enzymes are transition-metal complexes; e.g., photosystem I and II have iron-sulfur and manganese-oxide clusters as their active centers. The large number of atoms in ligands makes high-level calculation of the whole molecule impossible. In this situation, quantum embedding is necessary. Note that quantum embedding is different from the hybrid quantum-classical simulation discussed in section 3.1. Here, we embed a strongly correlated subspace in a single electron space. So, we need a single-particle theory for the whole molecule and a many-body theory for the correlated subspace, which is a small but functional part of the molecule. One embedding scheme utilizes density functional theory (DFT) as the single-particle theory and does the embedding via dynamical mean-field theory (DMFT) (Georges et al., 1996). In DMFT, the correlated subspace is referred as an impurity and the impurity problem is solved by an impurity solver. We can use unitary coupled-cluster theory, which can be run on quantum computers, to create an approximation of the ground state of the active region of the molecule. Then additional qubits are employed to represent the self-consistent bath that the impurity is coupled to. Time evolution then allows the Green's function to be determined for the impurity, which can be directly measured and have its self-energy extracted after the Green's function data is transformed from the time domain to the frequency domain. The impurity self-energy is then approximated as the self-energy of the molecule in DMFT, and we can use it to obtain the molecular interacting Green's function, which in turn will be used to calculate physical properties of the molecule. Most likely, these problems will require an inhomogeneous DMFT approach, with separate impurity problems for the different atomic sites in the strongly correlated material. The challenging computation of the local Green's function for each atomic site from the local self-energy would be carried out on a classical computer.

The impurity model is defined, in part, from the on-site Coulomb interaction *U*-matrix. One reliable way to determine these parameters from first principles is the constrained Random Phase Approximation (cRPA) method (Aryasetiawan et al., 2004, 2006). One aims to estimate the screened Coulomb interaction for selected bands of interest, that is, within a specified energy window. For this purpose, the particle-hole polarization between all possible pairs of occupied and unoccupied states is taken into account. This approach uses the Random Phase Approximation (RPA) and directly calculates the particle-hole polarization (Petersilka et al., 1996; Aryasetiawan et al., 2004).

To make DFT+DMFT fully *ab initio*, the hopping parameters and the Coulomb interaction parameters should be provided from first principles (in the DFT part of the calculation). The Hubbard Hamiltonian can be written as

(1) $$\begin{array}{r} {Ĥ}_{\text{Hubbard}}=\underset{i,j}{\sum }t_{ij}{ĉ}_{i}^{†}{ĉ}_{j}+\underset{i,\alpha ,\beta ,\gamma ,\delta }{\sum }U_{i,\alpha \beta \gamma \delta }{ĉ}_{i,\alpha }^{†}{ĉ}_{i,\beta }^{†}{ĉ}_{i,\gamma }{ĉ}_{i,\delta }. \end{array}$$

The hopping matrix *t*_*ij*_ comes from the DFT eigenenergies and provides the bath Green's function in DMFT. The Coulomb interaction *U*_*i*, α*βγδ*_ comes from the cRPA calculation described above, which is the only fully quantum-mechanical way to obtain the Coulomb interaction parameters. With the bath Green's function and *U* at hand, the effective action of the impurity problem is constructed. DMFT solves for the impurity Green's function by direct numerical sampling of the Green's function $G_{ij,\sigma }\left(t\right)=-\langle T{ĉ}_{i,\sigma }\left(t\right){ĉ}_{j,\sigma }^{†}\left(0\right)\rangle$. A classical computer algorithm often uses the continuous time quantum Monte Carlo algorithm (CT-QMC) (Gull et al., 2011; Zhang et al., 2019).

It has been proposed (Bauer et al., 2016) that a quantum computer algorithm could replace the CT-QMC calculation and provide the impurity Green's function *G*_*ij*, σ_(*t*), especially in cases where the classical computation suffers from the sign problem. Such a calculation embeds the impurity solver onto the quantum computer (quantum computing task), while the remainder of the DFT+DMFT iteration is carried out on classical computers. However, because describing the bath for the impurity problem is complex, it might be fruitful to instead simply solve the many-band lattice problem directly on the quantum computer. Indeed, this latter approach is more likely to be generalizable to large molecular systems. Of course, because molecules are not periodic, one will likely need to use inhomogeneous DMFT approaches if one takes the impurity problem approach.

**Figure 6:**
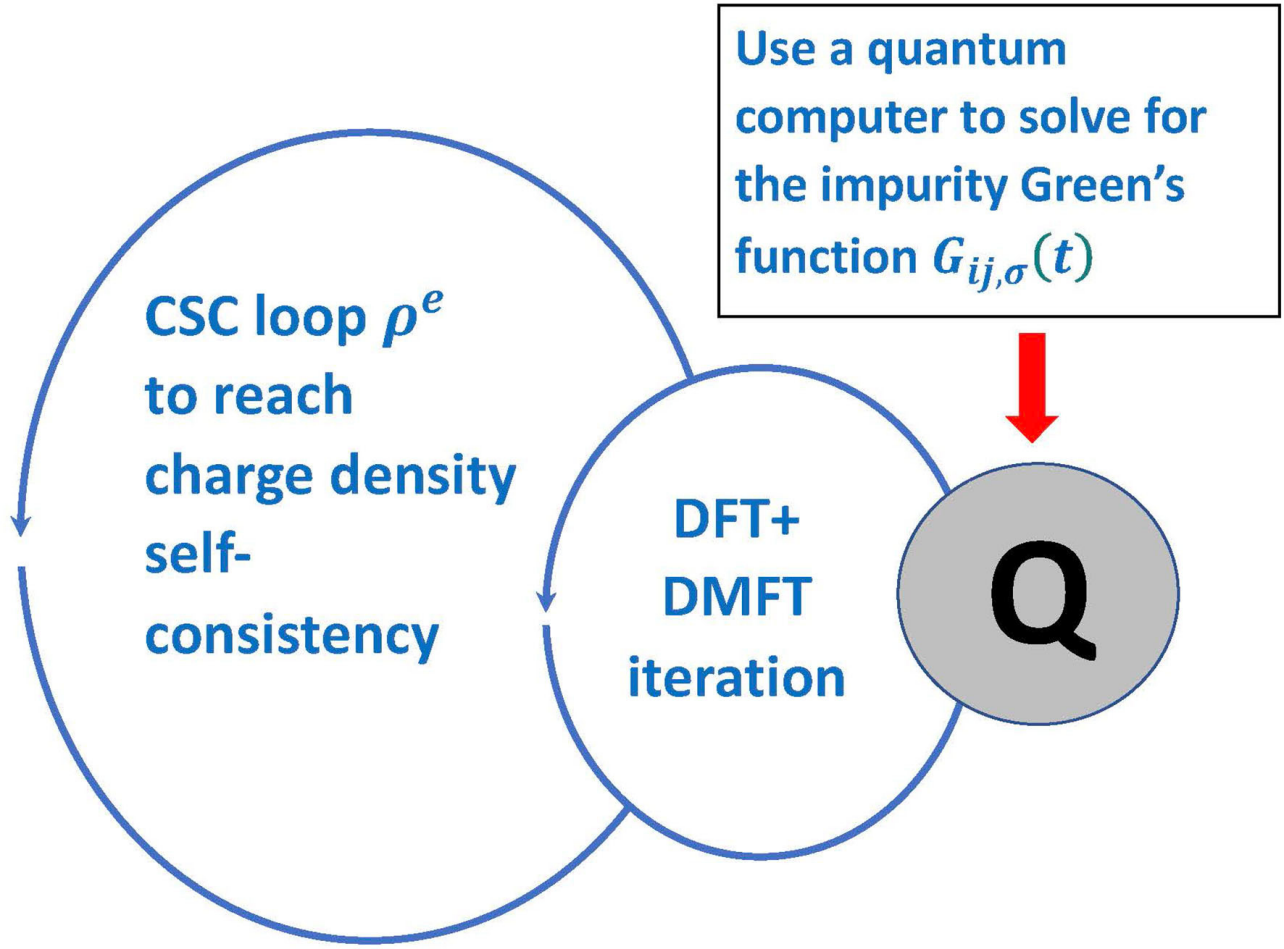
Schematic plot of the charge density self-consistent loop in conjunction with DFT+DMFT.

It often is important to embed the DFT+DMFT iteration into a larger loop of charge-density (ρ^*e*^) self-consistency (CSC) (Figure 6). It is known that CSC DFT+DMFT is necessary to capture charge density re-distributions even for very simple transition metal oxides like *V*_2_*O*_3_ under ambient conditions (Leonov et al., 2015). Similarly, in molecular calculations, charge redistribution is important for any simulation involving catalysis or other reactions. It is clear that all of these types of calculations must continue to be done on a classical computer. Only the complex strongly correlated part, involving the measurement of the Green's function will be done on the quantum computer. Because it can be directly measured, the connection between the quantum and classical calculation is simple to implement in this case.

## 4. Brief Overview of Chemistry on Quantum Computers

### 4.1. Quantum Algorithms and Methods

One basic challenge in computational chemistry is finding a way to avoid having to explicitly maintain the full many-body wave function, because the classical resource requirements of doing so grow exponentially with the system size. In a quantum computer, on the other hand, this scaling is linear in the number of qubits used. Another classical computational challenge is propagating the wave function in time by computing $e^{-iHt}|\Psi \rangle$. This calculation can also be efficiently implemented, in principle, on a quantum computer. This gives the promise of eventually being able to use a quantum computer to handle systems that cannot be feasibly calculated on classical computer. Such analysis can even be extended to beyond Born-Oppenheimer effects by including additional orbitals for the nuclei (Veis et al., 2016), but those approaches require significantly more resources and are likely to be applied to these large systems only far into the future. The disadvantage of working on the quantum computer is that the wave functions cannot be directly retrieved—either we resort to multiple calculations and measurements to obtain statistical knowledge of the wave function, or we settle for measuring some property of the wave function. Thus quantum algorithm design is not trivial.

In the last 20 years, significant progress has been made toward the goal of performing quantum chemistry on quantum computers (Cao et al., 2019). Most recently, Google has achieved a milestone in computational quantum chemistry by performing a Hartree-Fock calculation, the foundational algorithm in the field, on a superconducting quantum computer (Google AI Quantum and Collaborators, 2020). In this section, we describe two paradigmatic algorithms. The first is the variational quantum eigensolver (Peruzzo et al., 2014; McArdle et al., 2020), which is viewed today as the best candidate for performing chemistry on so-called noisy intermediate-scale quantum (NISQ) hardware. We also discuss the more accurate quantum phase estimation algorithm (Kitaev, 1995), which will ultimately emerge as the gold standard for quantum chemistry on a quantum computer because it can compute ground-state energies with only small systematic errors.

Of course, the first chemical systems put onto quantum computers are not going to be large biological molecules. But, with the development of the right algorithms for embedding, hierarchical structuring, and low-depth circuits, one might be able to advance biological science sooner than later. At the least, we should position ourselves to be able to try.

#### 4.1.1. Variational Quantum Eigensolver

In the NISQ era, quantum computers will not be able to accurately execute deep circuits. They also generate results that require error/noise mitigation due to errors in state preparation and measurement, and from infidelities in quantum gate executions. Within this realm of quantum hardware, there is an algorithm that shows great promise—the variational quantum eigensolver algorithm (Peruzzo et al., 2014). This algorithm is essentially a “state preparation and then measure” algorithm leading to low-depth circuits governed primarily by the complexity of the state preparation. One starts from a single reference state (usually the Hartree-Fock state) and then creates a variational ansatz that depends on a set of variational parameters. There are several options for how to do this, discussed below. Extensions and generalizations of the algorithm to determine excited states, which are important in many biological processes, have been developed such as to maintain the low-depth characteristic so important for the ability to run on NISQ systems (Higgott et al., 2019; Nakanishi et al., 2019).

However the wavefunction has been prepared on the quantum computer, we next need to measure the expectation value of the Hamiltonian to complete the calculation. The Hamiltonian is a Hermitian operator rather than a unitary one, so it cannot be evaluated directly on the quantum computer. Instead, we break it up into a sum of its mutually commuting unitary components and evaluate the expectation value of each unitary—the total expectation value is found by accumulating the total of all of the terms. As the number of orbitals increases, the number of terms in the Hamiltonian also increases. To date, only quite simple molecules have been computed on available quantum hardware (usually with minimal bases). The first approach was hydrogen and other simple binary and tertiary molecules (Kandala et al., 2017). More recently, the more complex system H_2_O in the STO-3G basis (Nam et al., 2020), has been handled.

Of course, this forms just the inner loop of the full variational calculation. One must now adjust the parameters in the variational wavefunction and repeat the whole process until the result converges with the minimum energy value. Because the data emerging from the quantum computer is noisy, this optimization problem requires complex algorithms on classical computers. The noise may even make it challenging to complete the calculation to the point where a true minimum can actually be located. The optimization problem may also suffer from “barren plateaus” which are large areas where the cost function gradient is extremely small. The sensitivity to noise can be reduced by calculating the derivative of how the energy changes when a variational parameter is changed from a matrix element measured directly on the quantum computer (Grimsley et al., 2019).

Because quantum computers have much slower clock cycles than classical computers, even with a quantum advantage for computing the results of a given measurement the quantum computations are expected to be slow. In addition, the parameters of a quantum computer often drift with time, creating additional issues associated with a changing accuracy for different expectation values over time. One may even need to correct for the drift over time or risk having data that is not accurate enough to be able to complete the outer loop of the variational cycle. Nevertheless, this approach remains the most promising approach available for now. Until we are able to perform extensive time evolution on a quantum system, it will remain the only viable strategy for quantum chemistry on NISQ era machines.

#### 4.1.2. Phase Estimation

The quantum phase estimation (QPE) algorithm was invented by Kitaev (1995) and is closely related to the quantum Fourier transform. It provides an alternative to solving the traditional eigenvalue problem H|ψ〉=E|ψ〉 on a quantum computer by transforming the problem to a unitary one and determining the phase λ*E* arising from the application of $e^{i\lambda H}$ to the eigenfunction as follows:

(2) $$e^{i\lambda H}|\psi \rangle =e^{i\lambda E}|\psi \rangle .$$

The λ parameter is introduced and a value chosen to ensure we can read the energy off without having the phase increase past 2π. We also need to measure enough binary digits in the number λ*E* to have an accurate measure of the energy. In addition, to get the energy corresponding to a particular eigenstate with high probability, one must prepare an initial state that has high overlap with that eigenstate. This could then involve a synergy with the variational quantum eigensolver algorithms in the following way: since the variational state is an approximation, it should have a high overlap with the true ground state, allowing it to be a good choice for the initial state that is used for the phase-estimation algorithm.

There are many benefits to the phase-estimation approach. First, it will give us an accurate estimate of the ground-state energy, with the accuracy determined by how many binary digits representing the phase are computed on the quantum computer. Second, it projects onto the eigenstate it measures. This allows it to also be employed as a state-preparation protocol; measuring the ground-state energy also has the consequence of preparing the ground-state wavefunction directly on the quantum computer where it can then be employed for further quantum computations. For example, if the embedding strategy for self-energy embedding theory (described in detail in section 5) is used, one can compute the zero-temperature Green's function directly from the ground-state eigenfunction after it has been prepared by QPE.

The challenge with phase estimation is that it requires us to be able to accurately perform time evolution. This is currently beyond the scope of available hardware and most likely we will need to wait for large-scale fault-tolerant quantum computers to be available to be able to carry out such computations. Nevertheless, it is important to think through how one would work with such an algorithm now, to be ready when such hardware becomes available. Also, sparse embedding theories will allow for time evolution sooner, and possibly even on NISQ machines.

#### 4.1.3. State Preparation

Both VQE and QPE require preparing an initial ansatz on the quantum computer. In other words, we start with an easy to initialize state, such as the Hartree-Fock state represented by |ψ_0_〉 = |0…0〉, and apply operations to transform that state to a representation of the desired wave function. The complexity of this step can be non-trivial. The ADAPT-VQE approach (Grimsley et al., 2019) dynamically constructs the ansatz by iteratively choosing operators from a pool of available operators. Another approach uses a unitary coupled cluster ansatz. In standard coupled cluster, the wave function has the form

(3) $$\begin{array}{r} \psi =e^{\hat{T}}\psi_{0} \end{array}$$

where $\hat{T}=\hat{T_{1}}+\hat{T_{2}}+\hat{T_{3}}+....$ represents singles, doubles, triples, etc. excitations relative to the Hartree-Fock ground state reference function. In principle, one can work out as many terms as computationally feasible. In conventional coupled-cluster theory, the operator is not unitary, but can be made unitary by letting $\hat{T}\to \hat{T}-\hat{T^{†}}$. Unitary coupled cluster is not practical computationally on classical computers, but is well-suited for quantum computers. A unitary singles and doubles coupled cluster approach will use a Trotterized form of the unitary coupled cluster ansatz (with only singles and doubles excitations in the exponent). Factorized forms of the unitary coupled cluster approach have also been considered, but usually, these approaches are not easily restricted to certain classes of excitations and might be better thought of within an ADAPT type methodology (Grimsley et al., 2019). There also are ansatzes that employ tensor-product-based wavefunctions (Cao et al., 2019).

## 5. Quantum Computer Embedding Strategies

The molecular systems of interest in biological processes are complex as well as geometrically extensive. It is also known that some processes depend crucially on small differences in structure and their associated energy differences. The timescales of processes may also span several orders of magnitude, from femtoseconds for molecular vibrational changes to milliseconds for some electron transfer processes and conformation changes. The challenge is that highly accurate calculations are needed for these extreme systems.

In addition, biological processes happen at finite temperature in a liquid environment, as opposed to many chemical processes that can be understood by studying the gas phase or materials structures that are often analyzed in isolation and at absolute zero. This means that a statistical description is needed to describe the full process and to obtain accurate reaction rates. Entropy and free energy play a crucial role.

Hence, biological molecules appear to be an ideal application for the promised power of quantum computing. However, with noisy intermediate-scale quantum computers (NISQ), such calculations are currently out of reach. Even when more fully fault-tolerant quantum computers become available, it is likely that the complete statistical quantum description of realistic biomolecular systems and processes will require decomposition of the system into parts, with the parts of the molecule involving the most demanding calculations done on the quantum computer and the less expensive calculations involving the rest of the molecule done on a classical computer. This section describes several such approaches which will necessarily be hybrid quantum-classical algorithms.

### 5.1. Quantum Computing on Fragments

In computational chemistry for large systems, the fragment molecular orbital (FMO) method (Gordon et al., 2012; Zahariev and Gordon, 2012; Tanaka et al., 2014) was developed to solve the problem described above: namely that the system is too large to treat directly as a whole. In that case, the molecule is divided into fragments that can be chosen to (in some sense) contain atoms that interact strongly with each other, but less strongly with atoms in other fragments. First, standard methods are used to obtain an accurate description of the isolated fragments. Then, other methods, also standard, are used to describe the interaction between the fragments and the effect those interactions have on the internal structure and properties of the fragments. The result converges to a solution for the complete system of all interacting fragments with controllable accuracy. In addition, the method shows linear scaling for large systems.

To describe the biochemical systems, one can envision a similar approach. Now, however, instead of using different methodologies for different regions, one uses a classical computer to describe one region and a quantum processor for the region where the model can use the advantage offered by quantum computing. The self-consistency would typically be carried out on a classical computer. The approach is similar to the VQE method described in section 4.1.1 for quantum chemistry on quantum computers: Part of the computation is performed on the quantum computer, some information is extracted from that calculation and handed to a classical computer, which then performs the next part of the computation. That computation results in new values to be used for the next iteration on the quantum computer.

For a hybrid description of a complex biological systems, the different parts of the computation are not only different stages in an algorithm, but also describe different spatial regions of the system. Let us call the region described on the quantum computer “primary” and the region described on the classical computer, which most often surrounds the primary region in space, the “environment.” We assume that the primary region fits in the quantum computer in the sense that it has sufficient qubits to represent both the quantum state in some encoding from fermions to spins (McArdle et al., 2020) and all the ancillary qubits necessary to execute the chosen algorithm.

It is necessary to choose quantum-mechanical methods to represent the states of both the primary and environment regions of the biological system so that the desired accuracy for the complete system can be achieved. It is not necessary that both regions are treated with the same method, as long as the physical description is consistent. The algorithm then inevitably requires that information is exchanged between the classical and quantum computers about the state description of the respective components. The classical computer can easily provide the necessary information to the quantum computer, which usually changes the state preparation on the quantum computer. However, as with VQE, obtaining accurate information about the state of the primary region as represented on the quantum computer can be challenging if the quantum state is complicated since there is no efficient way to directly access the entangled wavefunction stored on the qubits in the quantum processor. If a process of measurement needs to be called, then accurate calculations may require unacceptably large numbers of repeat runs of the program to obtain the required accuracy.

The general algorithm works as follows.

1. Specify a computational chemistry model for the environment region and initialize its state.
2. Specify a computational chemistry model for the primary region and prepare its state using the environment state parameters as needed.
3. Perform the algorithm to solve the computational chemistry model for the primary region on the quantum computer.
4. Extract the required information from the state of the primary region to perform the next iteration of the algorithm to converge the environment.
5. Perform the classical part of the algorithm for the primary region, using state information of the environment as needed.
6. Using information obtained by the classical part of the algorithm for the primary region, re-prepare the quantum computer for the quantum part of the algorithm for the primary region.
7. Repeat until the defined convergence criterion is met.

### 5.2. Sparse Green's Function Embedding Schemes

One of the challenges with accurate quantum chemistry calculations is that a large percentage of the correlation energy arises from the sum of many small contributions. This arises in part, because any standard orbital basis results in fairly full single-particle and two-particle interaction matrices. Hamiltonian evolution, or even variational methods require evaluating many, many terms. On a quantum computer, this leads to high-depth circuits, which become difficult to run on NISQ machines and may even be problematic on the expected fault-tolerant ones. One way around this problem is to transform the problem into a representation that is more sparse, or even to approximately force it into an extremely sparse representation. This is the idea behind the self-energy embedding theory (Tran et al., 2018).

Starting from an inexpensive classical calculation (such as Hartree-Fock plus MP2), one computes a representation for the self-energy of the full chemical system. Next, one determines the high-frequency moments of the self-energy. For the retarded Green's function, these moments of the self-energy are often determined by parameters in the Hamiltonian itself (the constant term is exactly determined from the Hartree-Fock approximation, the zeroth moment from the interaction, the first moment involves a few two-particle correlation functions, and so on). The strategy is then to construct an extremely sparse interaction for the effective Hamiltonian. This has the full single-particle contributions, but restricts the Coulomb interaction to on-site direct or exchange interactions only. These interaction terms are chosen to require that the low-order moments are preserved in the effective model. Then, one solves for the full self-energy of the effective model and then uses the effective self-energy as the self-energy for the full system. This approach guarantees that the low-energy moments of the final description of the molecule are exactly preserved. A self-consistency scheme is employed to update the approximation, as the moments depend on some expectation values which change as the Green's functions change with each iteration of the calculation. We also note that equality of low-order moments also implies that the two Green's functions agree exactly for short times.

The way we envision using this on a quantum computer for large molecules is to apply this approach to the strongly correlated core (or strongly correlated fragments) and one ends up with a much lower depth circuit for the time evolution because the Hamiltonian is so much sparser. This will allow more complex systems to be simulated earlier than possible with algorithms that include the full chemical complexity. The quantum computer simulates only the sparse Hamiltonian and determines the Green's function (or self-energy), which then is sent to the classical computer for the remainder of the algorithm. Even in the future, when fault-tolerant quantum computers become available, methods like the self-energy embedding theory will remain valuable as they can significantly streamline the number of operations needed to be run on the quantum computer.

## 6. Challenges

There are a few challenges associated with the modeling of biochemical systems. The first challenge is the accuracy required to describe the structures and processes. The standard is 1 kcal/mol or 4 kJ/mol, which in atomic units used in quantum mechanics is equal to 27.2 meV or 1 mHartree. Given that the energies of large molecules relevant in biochemistry are in the thousand Hartree range, the energies need to be calculated with a precision of 6 to 8 digits, which corresponds to a single precision IEEE floating point number on a classical computer. There are a large number of integrals with weights that are small. The contribution from each integral is small, but the sum adds up to a non-negligible contribution to the total energy. Because these numbers are obtained by a large number of floating point operations in the classical part of the computation, the minimum precision needed to perform this classical part of the calculation with controlled rounding error is 15 digits, which corresponds to the double precision floating point number on classical computers. For large molecules, relevant to biology, the integral contributions are sorted and added with small numbers first to build larger numbers that can be meaningfully added together to avoid critical round-off errors. That means that the step in the hybrid quantum-classical algorithm where values must be measured from the state in the quantum processor, these results need to be obtained with the right precision. Because the standard deviation of statistical sampling with *N* trials goes like $1/\sqrt{N}$, the number of measurements for a given accuracy ε is *N* = ε^−2^. A careful analysis is needed on what precision will be needed for the various terms to get acceptable accuracy for the total energies, because the required precision directly impacts the number of measurements that will be required, with a quadratic impact on total run time. Some further research to improve the algorithm for processing of the integrals will be needed to determine the minimum precision of each wave function component that must be combined with each integral to get the correct precision of the final result.

The second challenge in biochemical structure and process analysis and design is that the systems are at some finite temperature. That means a statistical description is essential. This has been taken into account for decades in the molecular dynamics simulations (Karplus and McCammon, 2002; Seabra et al., 2007; Salomon-Ferrer et al., 2013), with the method of replica exchange being one of the leading approaches (Roe et al., 2008).

However, chemical accuracy may not be sufficient. Decades of research to design drugs, enzymes, and catalysts has not been as successful as once hoped. A possible root cause is that chemical accuracy is insufficient to distinguish the competing mechanisms from each other, especially once the proper statistics at room temperature are taken into account. If 1 kcal/mo were adequate, scientists should have made more progress in identifying new mechanisms. To make the computations really insightful, it is likely that at least one and probably two or three orders of magnitude higher accuracy is required to generate new insights into drug, enzyme, and catalyst activities and reaction mechanisms.

These considerations make it clear that biochemical structures and processes are a fertile ground of problems to use and demonstrate the advantage of quantum computing over classical computing. It also shows the road to success will be difficult. But it promises to be wonderful journey!

## 7. Conclusion

This short review leaves us hopeful, but with many unanswered questions. It is clear that there are significant challenges that must be met before we can reap the benefits of quantum computers for biochemical applications. Nevertheless, due to the complexity involved in properly partitioning the sub units of these problems and then combining the results together, we need to start now to properly plan for how this will work. We can bring in ideas from a number of different areas where similar “divide and conquer” approaches have been tried and successfully completed. But the strategies that employ quantum co-processors to handle the most difficult parts of the calculations need to be properly thought out and structured so we can make rapid advances once the hardware is available. We did not map out a complete plan for how one can proceed. Instead, we described the different strategies that need to work together to achieve this goal. We are looking forward to seeing how everything comes together and how quantum computation will yield important and significant impacts on biochemistry.

## Author Contributions

CL wrote the biochemistry section. H-PC wrote the multi-scale simulation section. ED wrote the fragment method and its extension to quantum-classical hybrid application and the challenges. JF and BS wrote algorithms. All authors worked on the overall structure of the paper.

## Conflict of Interest

The authors declare that the research was conducted in the absence of any commercial or financial relationships that could be construed as a potential conflict of interest.
